# Does the revised intubating laryngeal tube (ILTS-D2) perform better than the intubating laryngeal mask (Fastrach)? – a randomised simulation research study

**DOI:** 10.1186/s12871-020-01029-3

**Published:** 2020-05-11

**Authors:** Thomas Ott, Katharina Tschöpe, Gerrit Toenges, Holger Buggenhagen, Kristin Engelhard, Marc Kriege

**Affiliations:** 1 Department of Anaesthesiology, Medical Centre of the Johannes Gutenberg University, Langenbeckstr. 1, 55131 Mainz, Germany; 2 Institute of Medical Biostatistics, Epidemiology, and Informatics, Medical Centre of the Johannes Gutenberg University, Mainz, Germany; 3 Rudolf-Frey Lernklinik Central Education Platform, Medical Centre of the Johannes Gutenberg University, Mainz, Germany

**Keywords:** [MeSH tree numbers]: simulation, Airway management [E02.041], Intubation [E05.497.578], Laryngeal masks [E05.497.578.475], Manikins [J01.897.280.500.545.129.400]

## Abstract

**Background:**

The intubating laryngeal tube (ILTS-D™) and the intubating laryngeal mask (Fastrach™) are devices that facilitate both extraglottic application and blind tracheal intubation. A revised model of the iLTS-D (for scientific reasons called ILTS-D2) has been designed but not yet evaluated. Therefore, we compared the ILTS-D2 with the established Fastrach under controlled conditions in a prospective randomised controlled simulation research study.

**Methods:**

After ethical approval, we randomised 126 medical students into two groups. Each participant received either Fastrach or ILTS-D2 to perform five consecutive ventilation attempts in a manikin. The primary endpoint was the time to ventilation in the last attempt of using the devices as extraglottic devices. Secondary endpoints were the time to tracheal intubation and the success rates.

**Results:**

There was no relevant difference between the two devices in the time to ventilation in the last of five attempts (Fastrach: median 14 s [IQR: 12–15]; ILTS-D2: median 13 s [IQR: 12–15], *p* = 0.592). Secondary endpoints showed a 2 s faster blind tracheal intubation using the Fastrach than using the ILTS-D2 (Fastrach: median 14 s [IQR: 13–17]; ILTS-D2: median 16 s [IQR: 15–20] *p* < 0.001). For both devices, the success rates were 100% in the last attempt.

**Conclusions:**

Concerning extraglottic airway management, we could not detect a relevant difference between the revised ILTS-D2 and the Fastrach under laboratory conditions. We advocate for an evaluation of the ILTS-D2 in randomised controlled clinical trials.

**Trial registration:**

Identifier at clinicaltrials.gov: NCT03542747. May 31, 2018

## Background

Extraglottic airway devices are recommended for health care providers who are inexperienced in tracheal intubation and as a rescue procedure in difficult airway situations [1]. However, tracheal intubation tends to show benefits in critically ill patients compared to extraglottic devises [2]. The Fastrach™ (FT-LM; Teleflex, Athlone, Ireland) intubating laryngeal mask is a well-described extraglottic ventilation device and is recommended for providers untrained in tracheal intubation [3]. The iLTS-D™ intubating laryngeal tube (VBM, Sulz a. N., Germany) is also used to facilitate a secondary tracheal intubation. First described in 2013, only a few studies have been published regarding its use [4, 5]. A revised version of the iLTS-D for scientific reasons called ‘ILTS-D2’ has been developed. Now, we evaluated this ILTS-D2 in an experimental setting using a manikin to prove its applicability [6]. In a prospective randomised controlled simulation research study, we compared the new ILTS-D2 with the FT-LM by using final year medical students as a representative sample of inexperienced airway providers with broad general medical knowledge. The primary endpoint was the time to ventilation when used as an extraglottic airway device in the last of five consecutive ventilation attempts. Our null hypothesis was that there is no difference in time to ventilation in the last of five attempts between the ILTS-D2 and the FT-LM when used as extraglottic devices.

## Methods

### Study design and time period

After ethical board approval (State Physicans’ Chamber of Rhineland-Palatinate, Registration Number: 2018–133,000-E., 18. May 2018), we conducted a randomised two-armed simulation research study with medical students as unbound samples. One group applied the FT-LM, and the other group applied the ILTS-D2 at a 1:1 ratio regarding the number of participants in both study groups. The study was conducted in our departmental simulation centre in June 2018.

### Participants

Medical students in their final year of medical school represent a sample with good theoretical knowledge but a lack of clinical experience and are therefore recommended for using extraglottic devices in emergency situations. A total of 126 medical students were recruited during their mandatory practical training periods.

### ILTS-D2

**Fig. 1:**
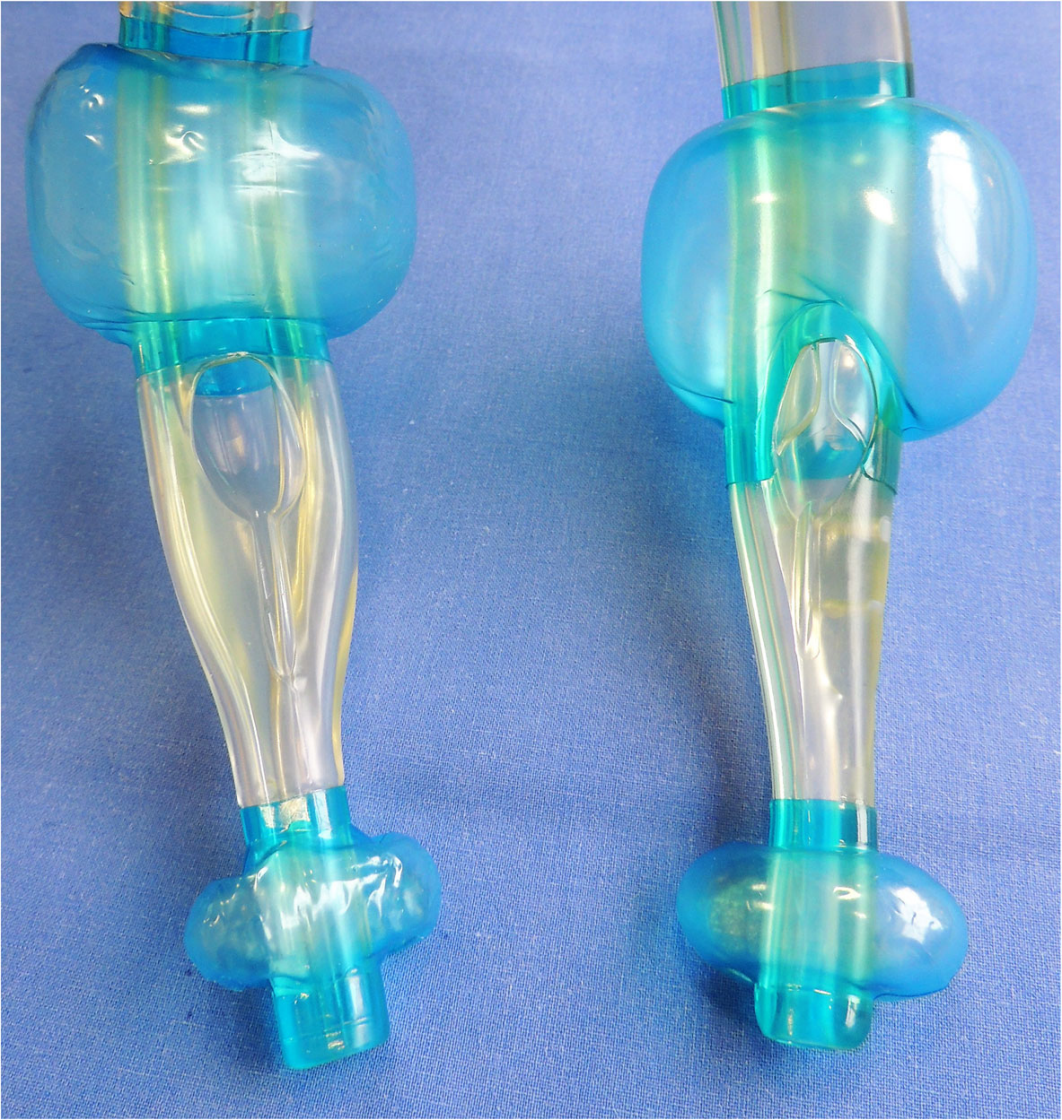
ILTS-D2 (left) and its precursor iLTS-D® (right) in direct comparison. Details are described in the main text

The revised ILTS-D2 differs from its predecessor in a number of regards [4, 5]; for the sake of clarity and conciseness, all cited lengths are measured from the tip (placed in the oesophagus entrance) along the convex frame of the device. The ILTS-D2 is 27 cm, thus 1 cm shorter than its predecessor. In both devices, the oesophageal cuff is similar, and the laryngeal orifice for ventilation and tube guidance is positioned from 6.5 cm to 9.5 cm from the distal margin. In comparison to the iLTS-D, the pharyngeal cuff of the ILTS-D2 is 2.5 cm more proximal and shorter in its longitudinal dimension (iLTS-D: 7.5–14.5 cm, ILTS-D2: 9.5–16 cm). Thus, the distance between the proximal and the distal cuff is longer in the ILTS-D2 than in its predecessor (iLTS-D: 6 cm, ILTS-D2: 7.5 cm). The ILTS-D2 is slightly wider between the cuffs than the iLTS-D (Fig. 1). Both devices fit sizes 4 and 5 of a classical laryngeal tube and indicate that the maximum size of tracheal tube that can be applied though the device is 8.0. A separately delivered 100 ml syringe for inflating the cuffs is colour coded according to the different sizes. A separately delivered tube can be inserted into the trachea through the laryngeal orifice that is shaped as a ramp. The angle of the tube guidance is approximately 35 to 40 degrees. The ILTS-D2 covers conventional laryngeal tube sizes 2 to 5 [7].

The ILTS-D2 is introduced along the hard palatinate until an elastic resistance indicates a proper fit in the oesophageal entrance. After inflation of the cuffs by the colour-coded syringe, extraglottic ventilation can be established. Then, the tube can be inserted through the device into the trachea.

### Fastrach™

The FT-LM is an established device evaluated for extraglottic and tracheal airway management [8]. We used the size 4 FT-LM with the provided tracheal tube.

### Manikin

We used the Laerdal Kelly™ ALS (Laerdal® Medical AS, Stavanger, Norway) manikin for this study. Manikins cannot completely replicate human anatomy. However, we have chosen this manikin because its airway offers one of the closest replications of the human anatomy for airway management simulation [9]. The manikin was placed on an emergency room stretcher, and the thorax was exposed.

### Data collection

After written informed consent was signed, the following demographic data were recorded: gender, age, and previous experience in airway management in patients: number of applications of 1) tracheal intubations, 2) laryngeal masks and 3) laryngeal tubes. Furthermore, professional qualifications such as paramedic, specialised nurses in anaesthesiology, medical clerkships and electives in anaesthesiology were protocolled. The participants were then randomised by flipping a coin to represent either the ILTS-D2 or the FT-LM. Each participant was familiarised with the particular device by a standardised introduction.

Only the allocated device was shown to the participant. The lubricated device with deflated cuffs, the appropriate tracheal tube, the syringe and the ventilation bag were placed on a table directly beside the manikin. First the extraglottic application then the tracheal intubation were explained by Peyton’s four-step approach: 1) demonstration in real time, 2) deconstruction (explanation and clarifying questions), 3) comprehension (explanation from participant to instructor), 4) execution by the participant [10]. The last step (execution) constituted the first of five attempts to place the device.

A total of five consecutive attempts were performed by each participant with one device. A stopwatch was started as soon as the participant touched the device and was stopped when the first chest rise was detectable [11]. After that, the stopwatch was started again as soon as the participant touched the tracheal tube and was stopped when the first chest rise was detectable. For both extraglottic ventilation and tracheal intubation, the time was not censored. However, censoring would occur due to the participants’ discontinuation. The instructors were not allowed to help during the attempts. If there was a chest rise, the attempt was counted as successful. If there was no chest rise or if the participant himself or herself discontinued the attempt, the attempt was counted as unsuccessful.

### Outcome measures

The primary endpoint was the time to ventilation in seconds (s) of extraglottic attempt five. Secondary endpoints were time to ventilation and intubation of all other attempts and success rates.

### Sample size

In March 2018, our sample size was determined by the actual number of students beginning the semester, 134, out of which 126 were recruited. The median time to ventilation using the Fastrach in our previous study (used as pilot data) for extraglottic ventilation was 20 s with a standard deviation of 5 s [4]. A reduction of 3 s was considered a clinically relevant difference in time for application of an airway instrument in the fifth attempt. With 63 participants per group, a difference in the medians of 3 s would have been detected with a power of 90.39% using a two-sided, level α = 0.05 Wilcoxon rank-sum test.

### Randomisation and blinding

Randomisation was performed by a coin flip (heads or tails) by two of the authors (TO and KT) before data collection. Heads was allocated to FT-LM and tails to ILTS-D2; this allocation was alternated every 20 participants. To gain an equal distribution of the level of previous experience, randomisation was stratified by professional qualification, clerkships and electives at a 1:1 ratio with regard to the devices. The participants could not be blinded to the devices for airway management because they had to apply the device.

### Statistical analysis

We used Microsoft Excel 2010 (Microsoft® Corporate Headquarters, Redmond, USA) and IBM SPSS Statistics Version 23 (IBM®, Ehningen, Germany). Considering that the data are non-normally distributed, data are shown as medians, interquartile ranges (IQRs), minima and maxima.

As no censored observations were present for the data of the primary endpoint (time to ventilation in attempt five of extraglottic application), the two groups were compared by a two-sided Wilcoxon rank-sum test. A *p*-value < 0.05 was considered statistically significant.

Regarding the secondary endpoints, censored observations were present for a few participants. Hence, group comparisons for the secondary endpoints were conducted either with a two-sided Wilcoxon rank-sum test if no attempts were discontinued by participants or with the log-rank test if attempts were discontinued by participants. *P*-values for secondary endpoints are given for exploratory reasons only.

## Results

### Primary endpoint: Extraglottic airway management

In the last of five attempts, no difference in time to ventilation between the FT-LM and the ILTS-D2 could be detected (FT-LM: median: 14 s (IQR: 12–15), ILTS-D2: median: 13 s (IQR: 12–15), *p* = 0.692) (Table 1).

**Table 1:** Time to ventilation in seconds for extraglottic application and tracheal intubation using the Fastrach and the ILTS-D2

		Median	IQR	Min / Max	P
Extraglottic application					
Attempt 1	Fastrach	19	16–22	12 / 30	
	ILTS-D2	17	15–21	11 / 70	0.183^a^
Attempt 2	Fastrach	16	13–17	11 / 34	
	ILTS-D2	15	13–18	10 / 43	0.982^a^
Attempt 3	Fastrach	15	13–17	10 / 25	
	ILTS-D2	15	13–17	10 / 25	0.647^a^
Attempt 4	Fastrach	14	13–16	9 / 21	
	ILTS-D2	14	12–16	9 / 27	0.846^a^
Attempt 5	Fastrach	14	12–15	8 / 20	
	ILTS-D2	13	12–15	9 / 28	0.592^a^,^c^
Tracheal intubation					
Attempt 1	Fastrach	19	17–22	14 / 41	
	ILTS-D2	23	20–26	16 / 160	< 0.001^a^
Attempt 2	Fastrach	16	15–19	12 / 32	
	ILTS-D2	20	17–22	13 / 140	< 0.001^b^
Attempt 3	Fastrach	15	14–18	11 / 33	
	ILTS-D2	18	16–21	12 / 168	< 0.001^b^
Attempt 4	Fastrach	15	13–18	11 / 35	
	ILTS-D2	18	15–22	12 / 114	0.002^b^
Attempt 5	Fastrach	14	13–17	10 / 27	
	ILTS-D2	16	15–20	12 / 112	< 0.001^a^

^a^ Wilcoxon rank-sum test; ^b^ Log-rank test; ^c^ primary endpoint

Time to ventilation in seconds for extraglottic application and tracheal intubation using the Fastrach™ and the ILTS-D2. Medians, interquartile ranges (IQRs), minima (Min), maxima (Max) and P-values of the particular statistics regarding participant-related censoring: Wilcoxon rank-sum test (attempts without censoring: all extraglottic attempts and intubation attempts 1 and 5), Log-rank test (attempts with censoring: intubation attempts 2, 3 and 4)

### Secondary endpoints: Extraglottic airway management and tracheal intubation through the device

In extraglottic application attempts one to four, as with the primary endpoint no difference in time to ventilation between the FT-LM and the ILTS-D2 could be detected. The time to ventilation continuously decreased from attempt one to five. All participants successfully established extraglottic application with both devices.

Tracheal intubation tended to be 4 s faster in attempt one and 2 s faster in attempt five using the FT-LM compared to the ILTS-D2 (attempt five: FT-LM: median: 14 s (IQR: 13–17), ILTS-D2: median: 16 s (IQR: 15–20), *p* < 0.001). The time to ventilation continuously decreased from attempt one to five (Table 1).

The success rates for extraglottic application over all attempts were 100%.

The success rates for tracheal intubation over all attempts was 98–100% with FT-LM and 95–100% with ILTS-D2, with a 100% success rate in both groups for attempt five (Table 2).

**Table 2:** Success rates. Success rates concerning extraglottic ventilation were 100% over all five attempts in both devices

Tracheal intubation		Success rate (%)	*n* success / *n* total	*n* missing
Attempt 1	Fastrach	100	63/63	0
	ILTS-D2	98	62/63	0
Attempt 2	Fastrach	100	63/63	0
	ILTS-D2	97	61/63	0
Attempt 3	Fastrach	98	62/63	0
	ILTS-D2	95	60/63	0
Attempt 4	Fastrach	98	61/62	1
	ILTS-D2	98	62/63	0
Attempt 5	Fastrach	100	63/63	0
	ILTS-D2	100	63/63	0

Success rates in percent (%) for the particular attempt exclusively concerning tracheal intubation. Number (*n*) of successful ventilations and total number (*n* total) of participants for the particular attempt. Number of missing data (*n* missing) for the particular attempt

### Participants

**Fig. 2:**
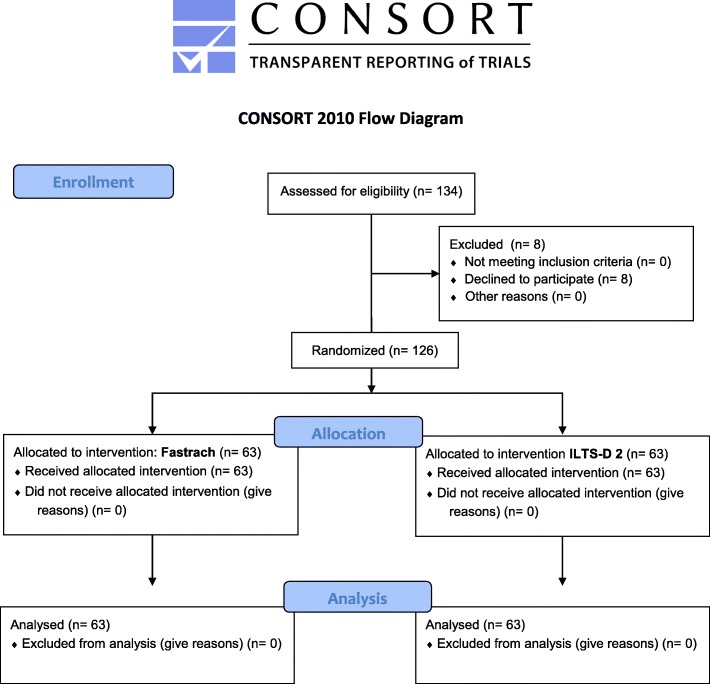
CONSORT flow chart

We obtained written informed consent from 126 participants from 28 May to 20 June 2018. A total of 63 participants were allocated to the FT-LM and 63 to the ILTS-D2 (Fig. 2). The median age was 27 years (FT-LM: median: 27 (IQR: 20–30), ILTS-D2: median: 27 (IQR: 26–30), *p* = 0.744) in both groups. Further details are cited in Table 3.

**Table 3:** Demographics of the participants

		Fastrach	ILTSD-2
		Number of participants (percentage of the sample^a^)	Number of participants (percentage of the sample^a^)
Age on years	median	27 (50%)	27 (50%)
Sex	female/male	30 (48%)/ 33 (52%)	40 (64%)/ 23 (37%)
Experience of tracheal intubation	None	31 (49%)	31 (49%)
	1–10	23 (37%)	26 (41%)
	11–50	8 (13%)	6 (10%)
	51–100	1 (2%)	0
Experience of laryngeal mask	None	31 (49%)	35 (56%)
	1–10	22 (35%)	16 (25%)
	11–50	10 (16%)	11 (18%)
	51–100	0	1 (2%)
Experience of laryngeal tube	None	49 (78%)	45 (71%)
	1–10	11 (18%)	10 (16%)
	11–50	3 (5%)	7 (11%)
	51–100	0	1 (2%)
Additional airway experience	None	41 (65%)	45 (71%)
	Anaesthesiologic clerkship	17 (27%)	15 (24%)
	Anaesthesiologic elective	4 (6%)	3 (5%)
	Anaesth. clerkship and elective	1 (2%)	0
Previous professional experience	None	45 (71%)	44 (70%)
	Emergency medical technician	4 (6%)	4 (6%)
	Paramedic	9 (14%)	13 (21%)
	Nurse	4 (6%)	1 (2%)
	Specialised anaesthestetic nurse	1 (2%)	0
	Special. anaesth. Nurse + paramedic	0	1 (2%)

^a^sum of percent occasionally exceeds 100 due to mathematical rounding

## Discussion

The present study shows that the revised ITLS-D2 as an extraglottic airway device is as easy to apply as the FT-LM in terms of efficacy in a simulated environment. The median time to ventilation was 14 s with the Fastrach™ and 13 s with the ILTS-D2. Secondary endpoints concerning extraglottic application tended to show no relevant differences in attempts one to four as well. Based on these findings, the revised ILTS-D2 is considered a well-applicable extraglottic airway device for inexperienced providers, at least under laboratory conditions.

However, the time to intubation tended to be longer for the ILTS-D2 than for the FT-LM. The pharyngeal cuff of the ILTS-D2 is positioned at a more cranial level of the pharynx in comparison to its precursor, which might cause a relative delay of intubation in comparison to the FT-LM. The ITLS-D2 is constructed for blind intubation but can also be combined with fibreoptic guided intubation through the ILTS-D. Undoubtedly, intubation endoscopy is the most reliable technique in this context, but it requires advanced skills and is, therefore, reserved for experienced airway management providers only [12]. However, the success rate using the ILTS-D2 was comparable with that of the FT-LM over all five attempts. Thus, the ITLS-D2 can be considered a stand-alone device for blind intubation under laboratory conditions, nevertheless fibreoptic control generally is recommendable for tracheal intubation in this context of extraglottic airway devices.

### Comparison to previous studies

The present study yielded timeframes that are valid for simulation research studies. The predecessor of the ILTS-D2 yielded timeframes as an extraglottic device of 14.5 s [4]. The time to ventilation using the FT-LM was in the range of 8 to 29 s [13–15] and as an intubation conduit was approximately 20 s [15, 16]. Furthermore, the FT-LM inheres broad evidence as a ventilation and intubation device. Therefore, in the present study, the FT-LM was used as a reference device [8, 17].

### Limitations

Simulation research lacks translation to real patients. In particular, evaluations of airway devices inhere alternations of reality due to the quality of tissue, absence of mucus and other anatomic aspects [9]. Furthermore, the simulated situation was strictly focused on airway management, not on any other medical aspects like emergency situations or insertion techniques of the particular device [1, 18, 19]. Nevertheless, it is recommended to train and evaluate providers with manikins due to the possibility of standardisation of situational and anatomy-related conditions [20]. Analogue to pharmacologic principals, the authors believe that airway devices ought to be evaluated under laboratory conditions before application in patients. Cook et al. noted this problem and recommended a structured analysis of novel airway devices, similar to the developmental process of drugs [21].

### Clinical implications

After the first attempt, both devices presented with an early reduction of time to ventilation. However, there was still a decrease in time to ventilation from attempt four to five. Therefore, we did not reach the end of a learning curve with five application attempts. These findings indicate that even under simulated conditions, more than five attempts may be necessary for inexperienced providers to reach proficiency with these devices.

## Conclusions

Concerning extraglottic airway management, we could not detect a relevant difference between the revised ILTS-D2 and the FT-LM under laboratory conditions. Concerning intubation, in our sample, participants intubated the trachea 2 s faster using the FT-LM than using the ILTS-D2; however, this amount of time was not relevantly different. Under these conditions, the authors advocate for further investigations in controlled clinical trials.

## Data Availability

The datasets generated and analysed during the current study are available in the Mendeley Data repository under Digital Object Identifier (DOI): https//www.doi.org: 10.17632/sj5362f4n7.1
